# Particulate matter and heavy metal deposition on the leaves of *Euonymus japonicus* during the East Asian monsoon in Beijing, China

**DOI:** 10.1371/journal.pone.0179840

**Published:** 2017-06-29

**Authors:** Tong Zhang, Yuxuan Bai, Xiuling Hong, Liwei Sun, Yujun Liu

**Affiliations:** 1 College of Biological Sciences and Biotechnology, Beijing Forestry University, Beijing, China; 2 Yanchi Research Station, School of Soil and Water Conservation, Beijing Forestry University, Beijing, China; University of Vigo, SPAIN

## Abstract

Plants can be effectively used as bio-monitors of environmental pollution. However, how the particulate matter (PM) and heavy metal retention ability of plants changes in different areas with human disturbance along with monsoon has not yet been investigated in urban ecosystems. In this study, we measured the amount of PM and heavy metals such as Ni, Cr, Cu, Pb, and Zn accumulated by the leaves of *Euonymus japonicus* during the East Asian monsoon from different functional units in Beijing, China. A rinse-and-weigh method developed in our laboratory was used to determine the mass of the PM, and electro-thermal atomic absorption spectrometry was used for heavy metal analysis. We found that the types of functional units had little influence, whereas the monsoon had a significant effect on the deposition of PM: northwest areas during the monsoon had the lowest effect (with 0.005, 0.453, 0.643, and 1.569 g/m^2^ fine, coarse, large, and total PM, respectively), and the southeast areas during the monsoon had the highest effect (0.015, 2.687, 1.941, and 4.228 g/m^2^ for fine, coarse, large, and total PM, respectively). Notable, we found considerable variations in heavy metal accumulation across the functional units analyzed, that is, the accumulation level was higher in communities than in parks (P < 0.0001 for all heavy metals). Moreover, a positive relationship was found between PM retention and heavy metal accumulation by the leaves of *E*. *japonicus*. Taken together, our results suggested that the PM and heavy metal retention ability of *E*. *japonicus* was sensitive to human disturbance and monsoon in Beijing. Since *E*. *japonicus* is a widely distributed tree and has the ability of to purify the atmosphere, it is an ideal plant for mitigating urban environmental pollution.

## Introduction

In China, rapid economic development has been accompanied by serious environmental issues. Urbanization and industrialization have led to the release of industrial waste gases and vehicle exhaust into the air, water, and soil [1,2]. Therefore, atmospheric pollution has attracted considerable attention; particulate matter (PM) in addition to heavy metals and other harmful components have been shown to have serious impact on human health [3]. Epidemiologic studies have revealed that long-term exposure to fine particulate air pollution is an important environmental risk factor for cardiopulmonary and lung cancer mortalities, and that improved air quality results in relatively prompt and sustained health benefits [4,5].

Beijing is the capital of China with a population of over 21 million, which has increased 1.6 times in the past 17 years. The increasing population has led to an increased number of vehicles and industrial activities, which frequently cause heavy air pollution, appearing as haze weather and mainly consisting of fine PM, and adversely affect public health [6–8]. Environmental pollution in Beijing is attributed to the following two factors. First, the emission of pollutants from anthropogenic sources is strongly correlated with severe air pollution [9]. The atmospheric source apportionment of PM of less than 2.5 μm (PM2.5) revealed that its main sources in Beijing are coal combustion, vehicle exhaust, industrial emission, and dust pollution [10,11]. Second, Beijing is located in the East Asian area (39°56ʹN and 116°20ʹE) on the northwest border of the North China Plain and is surrounded by the Yanshan Mountains. The unique topography of Beijing hinders the diffusion of atmospheric pollutants. Moreover, in addition to human activities and unique topography, the variation of fog and haze days is also highly correlated with meteorological factors such as temperature and precipitation [12,13]. However, studies on anthropogenic factors and environmental factors are still insufficient, especially on PM and heavy metals.

As the most active atmospheric system, considerable attention has long been paid to the East Asian monsoon. The climate in China is mainly controlled by the East Asian monsoon, and changes in weather and climate are significantly impacted annually [14–16]. Beijing belongs to the sub-humid warm temperate continental monsoon climate. In winter (the East Asia winter monsoon), cold and dry winds from Siberia prevail over Beijing from December to February, which lead to the preference of Northwest and North winds. In summer (the East Asia summer monsoon), wind from Pacific Ocean and Indian Ocean blow to the continent of Asia, which lead to the preference of Northwest winds [17]. The increasing haze weather has been thought to be related to the East Asia monsoon. A study investigated the inter-annual variation of the wintertime fog-haze days across China from 1972 to 2014, as well as its relationship with East Asian winter monsoon. They found that the wintertime fog-haze days across China have a close relation with East Asian monsoon [13]. Nevertheless, Beijing is characterized by the East Asian monsoon, and the temperature, precipitation, and general atmospheric circulation, and thus the formation, emission, and transportation of PM, are known to be significantly influenced by monsoon. Hence, investigating the interaction between pollution and monsoon and identifying a suitable species for future afforestation would be valuable [18].

Accumulation of PM, in particular that associated with heavy metals, in the atmosphere poses a potential threat to human health [19,20]. Trees have the ability to capture PM and accumulate heavy metals in an environmentally friendly manner. Several studies have investigated the application of vegetation for environmental protection [21,22]. Plants in rural areas were found to improve the local environment, including temperature, humidity, and air quality [23,24]. A shelter-forest belt was shown to have a dust-retention rate of 38.9%–46.1% [25]. The number of particles per leaf per centimeter square for five evergreen species was shown to be 1.95 × 10^7^ [26]. In New York, the total amount of PM2.5 removed annually by trees was found to vary from 4.7 tons in Syracuse to 64.5 tons in Atlanta, with annual PM removal costs ranging from $1.1 million to $60.1 million, respectively [24]. In urban areas, heavy metals are primarily released via atmospheric emissions. Inorganic elements and organic compounds, especially heavy metals and polyaromatic hydrocarbons, tend to adhere to PM to form fine particulates and dust that finally deposit on land, as well as on trees [27,28]. By transferring elements from the abiotic to biotic environment [29], tree leaves and roots absorb many particulates containing hazardous materials present in the atmosphere or that deposited on land, respectively. *Betula pendula* density was found to be positively influenced by traffic intensity [21]. Scots pine [30], chestnut [31], Mediterranean oak species (*Quercus ilex*) [32], as well as several ornamental plants [33], were used to detect heavy metal pollution. Thus, trees can be considered to play important roles in purifying the environment and fixing heavy metals [34].

The dry and cold conditions in Beijing are not suitable for the growth of deciduous broadleaf plants. Unlike deciduous species, *Euonymus japonicus* is an evergreen shrub, has a strong adaptive ability to the environment, and can efficiently reduce PM in winter. This species is important for cities located in the northern regions such as Beijing. From the macroscopic point of view, *E*. *japonicus* shows rapid growth; is pollution-, drought-, disease-, and heat-resistant; and is widely distributed [35,36]. From the microscopic perspective, it has a smooth leaf surface and thick wax layer; the stoma are concave, increasing their ability to capture PM [37]. Zhao et al. [38] investigated the ability of four types of typical trees to capture PM and found that *E*. *japonicus* showed a sensitive response to atmospheric PM. Moreover, the roots, leaves, and stems of *E*. *japonicus* have strong ability of enriching heavy metals [39]. No significant relationship has been found between the heavy metal concentrations on the leaves of *E*. *japonicus* and those in the soil. Air pollution caused by anthropogenic activities are considered to be the main source of heavy metals in the leaves of *E*. *japonicus* [40]. Hence, *E*. *japonicus* is a good indicator plant for plant-pollution interactions and an outstanding afforestation species, which is sensitive to heavy metal accumulation and PM retention.

Although plants can be effectively used as bio-monitors of environmental pollution, the variations in pollution items (e.g., PM retention and heavy metal accumulation) on plants according to the direction of monsoon has not yet been investigated. Considering the wide distribution and resistance property of *E*. *japonicus*, we investigated the ability of its leaves to accumulate PM and heavy metals by collecting samples from various locations in Beijing. This study aimed to investigate the influence of monsoon direction and environmental factors on the ability of *E*. *japonicus* leaves to capture PM and heavy metals.

## Material and methods

### Study area and sample collection

No specific permissions were required for all the locations and activities in our study in that we have conducted our research with no destroys to the trees, and the species is not an endangered or protected species.

As mentioned above, the climate over Beijing is mainly controlled by the East Asian monsoon; therefore, based on the local climate, we selected sampling areas along this monsoon direction, namely, from northwest to southeast in the downtown area of Beijing.

**Fig 1:**
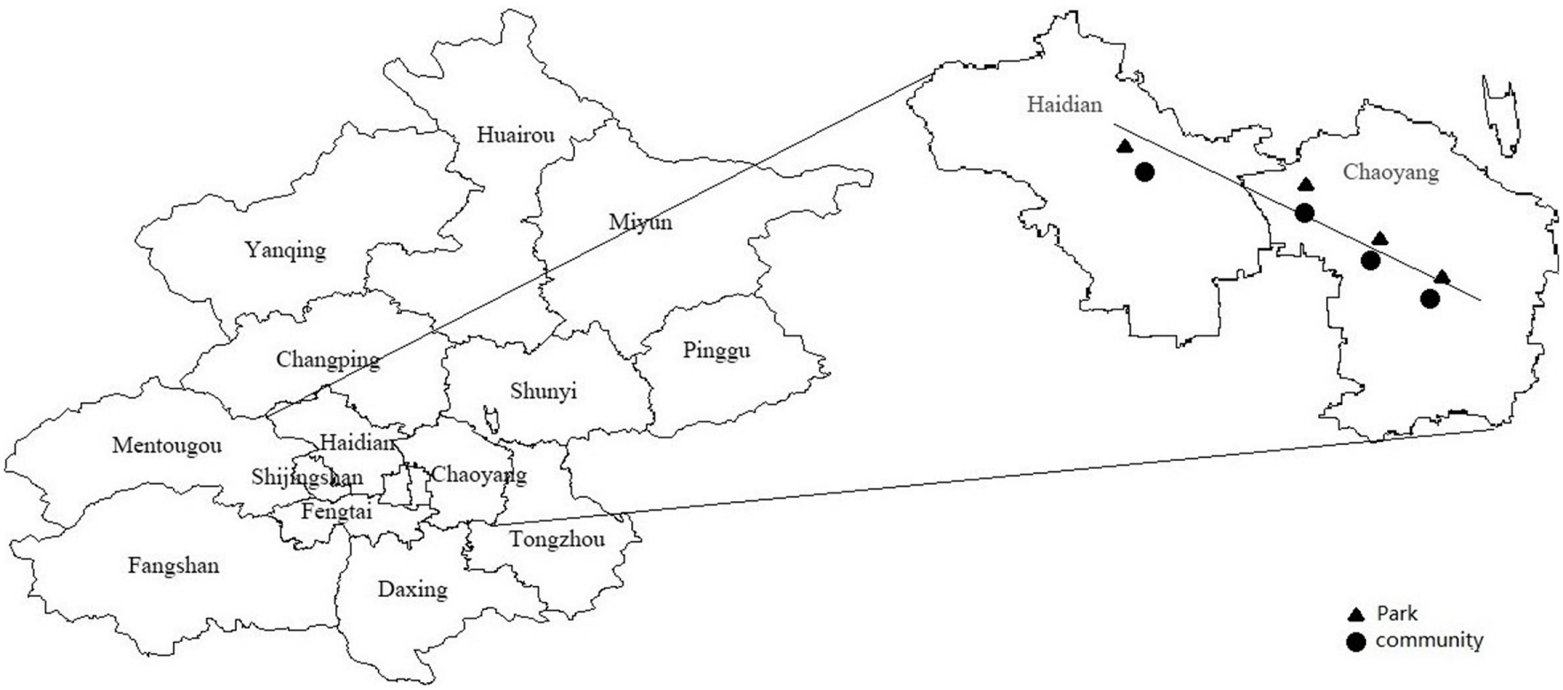
Location of the sampling sites in Beijing. The left panel shows the map of Beijing; Haidian and Chaoyang Provinces in the downtown areas are enlarged. The sampling sites are marked in the downtown areas along the monsoon line from Northwest to Southeast. These sites include 4 communities and 4 parks. The 4 communities are as follows: west campus of China Agricultural University (C1), Chinese Academy of Sciences (C2), Beijing University of Chemical Technology (C3), and King Stone Apartment (C4); the 4 parks are as follows: Medicinal Plant Development (P1), Olympic Forest Park (P2), Yuan Dynasty City Wall Relics Park (P3), and Chaoyang Park (P4).

In all, eight sampling sites were selected as shown in Fig 1; they included the following four communities: the west campus of China Agricultural University (C1 40°03ʹN and 116°27ʹE), Chinese Academy of Sciences (C2 40°02ʹN and 116°39ʹE), Beijing University of Chemical Technology (C3 39°98ʹN and 116°36ʹE), and King Stone Apartment (C4 39°94ʹN and 116°48ʹE); and the following four parks: Medicinal Botanical Gardens of the Institute of Medicinal Plant Development (P1), Olympic Forest Park (P2), Yuan Dynasty City Wall Relics Park (P3), and Chaoyang Park (P4). There are no polluting factories and high way within 5km. In fact, the polluting factories are retreat from Beijing since 2015, and basicly, there is no polluting factories in the main district.

All sampling activities were performed in January 2015, when no snow occurred, and the wind scale was below five over ten consecutive days before sampling. At each sampling site, 3 trees were chosen for sampling; in total, 24 trees were sampled. The sampled trees had a height between 1.2 and 1.5 m and were almost of the same age. Considering that differences in the height of sampling trees might cause variations in results, each tree was sampled at a height of 1.0 m. The sampled trees were healthy (i.e., without chlorotic or necrotic symptoms). Leaves (fresh weight, 50 g) were collected from four directions of each sampling tree, and the leaves collected from the same sampling area were mixed together. The samples were placed in paper bags and brought to the laboratory. Plastic gloves were used during sampling to avoid contamination.

### Measurement of PM

The PM analysis was conducted using a rinse-and-weigh method developed in our laboratory [41]. Briefly, the collected leaves were soaked in distilled water for 60 min, and PM fractions of three categories (large, coarse, and fine) were rinsed off from the leaves by using 200 mL distilled water and a banister brush. The samples were cleaned three times to ensure the complete removal of the leaf-deposited PM, and the rinsed water (around 600 mL) was pooled and weighed (*M*_T1_, ±0.001 g). The total liquid was agitated using a magnetic stirrer. Next, 50 mL of the stirred liquid was placed in a pre-weighted petri dish and evaporated to dryness in an oven (STIK Ltd. US) at 40°C. Samples were weighed before (*M*_p1_, ±0.001 g) and after (*M*_p2_, ±0.001 g) the drying procedure. The total suspended PM (*M*_T2_ ±0.001 g) was calculated as follows.

$$M_{\text{ T}2\;}=M_{\text{ p}2}\times \frac{M_{\text{ T}1}}{M_{\text{ p}1}}$$ (1)

The remaining stirred liquid (around 550 mL) was filtered sequentially by using 10- and 2.5-μm filters (Millipore, USA), which allowed capturing of large (*M*_s>10_) and coarse (*M*_s2.5–10_) PM, respectively. Before and after the samples were weighed, the filters were dried for 2 h at 60°C and stabilized over 24 h in a glass desiccator. The total amount of large, coarse, and fine PM (*M*_pm>10_, *M*_pm2.5_, *M*_pm10_) in the turbid liquid was calculated using Eqs (2), (3) and (4), respectively.

$$M_{\text{ pm>}10}=M_{\text{ s>10}}\times \frac{M_{\text{ T1}}}{M_{\text{ T1}}-M_{\text{ p1}}}$$ (2)

$$M_{\text{ pm}2\text{.}5-10}=M_{\text{ s}2\text{.}5-10}\times \frac{M_{\text{ T1}}}{M_{\text{ T1}}-M_{\text{ p1}}}$$ (3)

$$M_{\text{ pm}2\text{.}5}=M_{\text{ T2}}-M_{\text{ pmm}2\text{.}5-10}$$ (4)

HP Scanjet 200 scanner was used to scan the leaf area, which was measured using Photoshop software.

### Measurement of heavy metals

For heavy metal analysis, three duplicate samples were analyzed for each site. Leaf samples were washed as described by Kozlov [42]. Paper bags containing leaves were placed at 105°C for 30 min to deactivate enzymes. The leaves were dried at 85°C in an oven to a constant weight. Dried samples were ground using a grinder (Xtreme Ltd., US) for 90 s at a 30-s interval, and then passed through a sieve (ψ = 0.25 mm); the dry powder was placed in polythene bags and stored in a desiccator.

Accurately weighed powder (1 g ± 0.0001 g dry weight each) was placed in an Erlenmeyer flask. Samples were digested using the HNO_3_-HClO_4_ method [3]. Briefly, 30 mL of mixed acid (the ratio of HNO_3_ and HCLO_4_ is 5:1) was added to each sample. The samples were then warmed using an electric stove until no N_2_ evolved, and the digested solutions became clear. The solution was filtered into a 100-mL volumetric flask for further analysis. The national standard of soil samples (GSS1-8) was used as the analytical quality control, and it was obtained from the National Institute of Metrology [3].

Heavy metals such as Ni, Cr, and Pb were analyzed using inductively coupled plasma optical emission spectroscopy (Optima 8000; Perkin Elmer Ltd.), with a characteristic mass of d = 0.2 pg and limit of detection of (3σ) = 0.01 μg/L. Cu and Zn were analyzed using an atomic absorption spectrophotometer (Spectr AA 200; VARIAN Ltd.) with sensitivity of 5 ppm Cu > 0.75 Abs and precision of <0.5% RSD. The standard reference material was obtained from the National Sharing Platform for Reference Materials.

### Statistical analysis

Firstly, Two-way ANOVA was used to test the influence of monsoon and functional areas and their combined effects on dust retention and heavy metal accumulation. Then we used one-way ANOVA to analysis the differences of retention and heavy metal accumulation along monsoon gradient. t-tests was used to compare the differences of retention and heavy metal accumulation between functional areas in each sampling site. Redundancy analysis was conducted to analysis the relationships among heavy metal accumulation retention and functional areas. t-test, one-way and two-way analyses of variance (ANOVAs) by using JMP SAS 11.0. Redundancy analysis was conducted using R vegan package. All data was analysis by normal distribution and homogeneity of variance analysis, which meet the requirements of variance analysis.

## Results

The mass of the three sizes of PM accumulated on *E*. *japonicus* leaf surfaces is shown in Table 1. The total suspended, large, coarse, and fine PM retained on *E*. *japonicus* leaves ranged from 1.569 to 2.201, 0.854 to 1.527, 0.453 to 2.687, and 0.005 to 0.015 g/m^2^, respectively, in community areas, and from 1.684 to 2.571, 0.643 to 1.941, 0.396 to 1.319, and 0.01 to 0.018 g/m^2^, respectively, in park areas. Our results showed that functional units did not influence the capture of large (*p* = 0.4983) and fine (*p* = 0.8317) PM (Table 2), whereas affected the retention of coarse and total suspended PM (*p* = 0.0053, *p* = 0.0359).

**Table 1:** The mass of surface particulate matter (PM; Fine, Coarse, Large, and Total suspended) accumulated by *E*. *japonicus* leaves at different sampling sites.

Site	Fine (g/m^2^)		Coarse (g/m^2^)		Large (g/m^2^)		Total (g/m^2^)	
	C	P	C	P	C	P	C	P
**1**	0.032 ± 0.016^a^*	0.008 ± 0.003^A^	0.453 ± 0.143^a^	1.319 ± 0.321^A^	1.234 ± 0.283^a^	1.243 ± 0.364^A^	2.201 ± 0.604^a^	2.571 ± 0.257^A^
**2**	0.005 ± 0.000^b^	0.010 ± 0.003^A^	0.761 ± 0.026^a^	1.139 ± 0.121^A^	0.854 ± 0.025^a^	0.643 ± 0.072^A^	1.569 ± 0.107^a^	1.792 ± 0.056^B^
**3**	0.011 ± 0.007^b^	0.016 ± 0.002^A^	0.671 ± 0.337^b^	0.545 ± 0.077^A^	1.234 ± 0.395^a^	1.123 ± 0.084^A^	1.919 ± 0.524^a^	1.684 ± 0.013^B^
**4**	0.015 ± 0.002^ab^	0.018 ± 0.005^A^	2.687 ± 0.218^b^	0.396 ± 0.072^B^	1.527 ± 0.240^a^	1.941 ± 0.174^B^	4.228 ± 0.271^b^	2.355 ± 0.098^A^

* Superscripted letters in different columns indicate significant differences as determined by one-way analysis of variance (*P* < 0.05)

**Table 2:** The influence of monsoon, functional sites, and their combined effects on the particulate matter (PM) accumulated by *E*. *japonicus* leaves.

PM	Monsoon		Function		Monsoon & Function	
	F	*P*	F	*P*	F	*P*
**Fine (0–2.5 μm)**	2.274	0.1069	0.474	0.4983	4.427	0.0135
**Coarse (2.5–10 μm)**	16.468	<0.0001	9.462	0.0053	49.457	<0.0001
**Large (>10 μm)**	9.898	0.0002	0.046	0.8317	1.177	0.3402
TSP*	17.150	<0.0001	4.965	0.0359	8.383	0.0006

* total suspended PM

**Fig 2:**
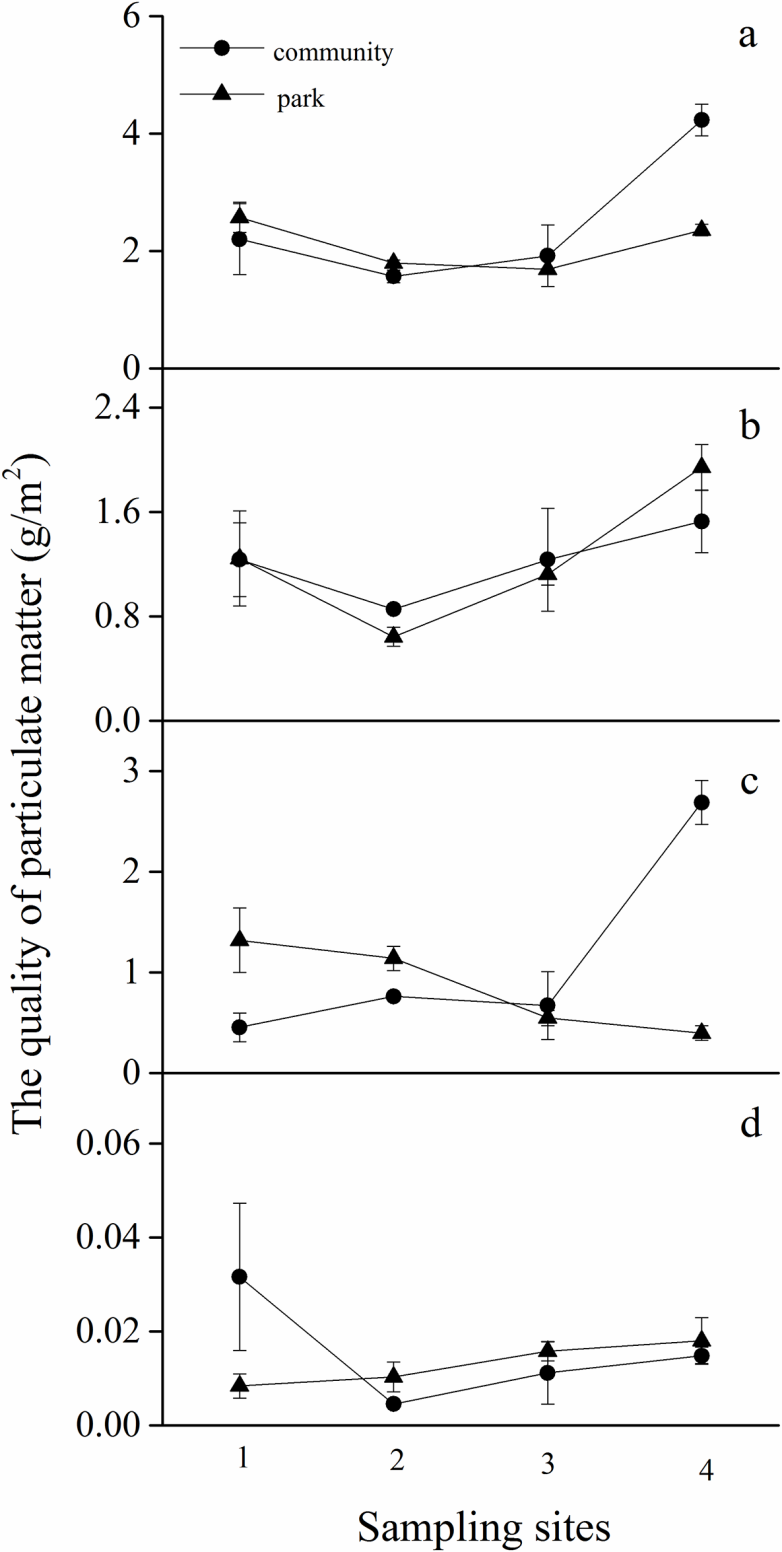
Four particulate matter (PM) fractions accumulated by *E*. *japonicus* across different sampling sites during monsoon in Beijing. (a) Total suspended particulates (b) large particulates (10 < diameter ≤ 100 μm), (c) coarse particulates (2.5 < diameter ≤ 10 μm), and (d) fine particulates (diameter ≤ 2.5, i.e., PM2.5); (c) + (d) = PM10.

Monsoon remarkably affected the capturing of total, large, and coarse PM (Table 2; *p* < 0.0001, *p* = 0.0002, *p* < 0.0001, respectively). More PM was retained on *E*. *japonicus* leaf surfaces during the monsoon (Fig 2). For community areas, the mean trend of total, large, coarse, and fine PM increased from the Northwest to Southeast, whereas the retention of total, large, and fine PM on site 1 was higher than that on site 2. Further, coarse PM retention was remarkably increased on site 4. In the park areas, the mean trend of total, large, and fine PM increased from the Northwest to Southeast, whereas the level of coarse PM decreased during monsoon. The levels of total and large PM were higher on site 1 than on site 2. Our results showed that all PM fractions, except large ones, were impacted by the mixed effects of functional areas and monsoon (*P* = 0.0135, *P* < 0.0001, and *P* = 0.0006 for fine, coarse, and total suspended PM, respectively). Thus, the effects of monsoon were greater than those of functional units on the capturing of PM on the surfaces of *E*. *japonicus* leaves (Table 2).

**Fig 3:**
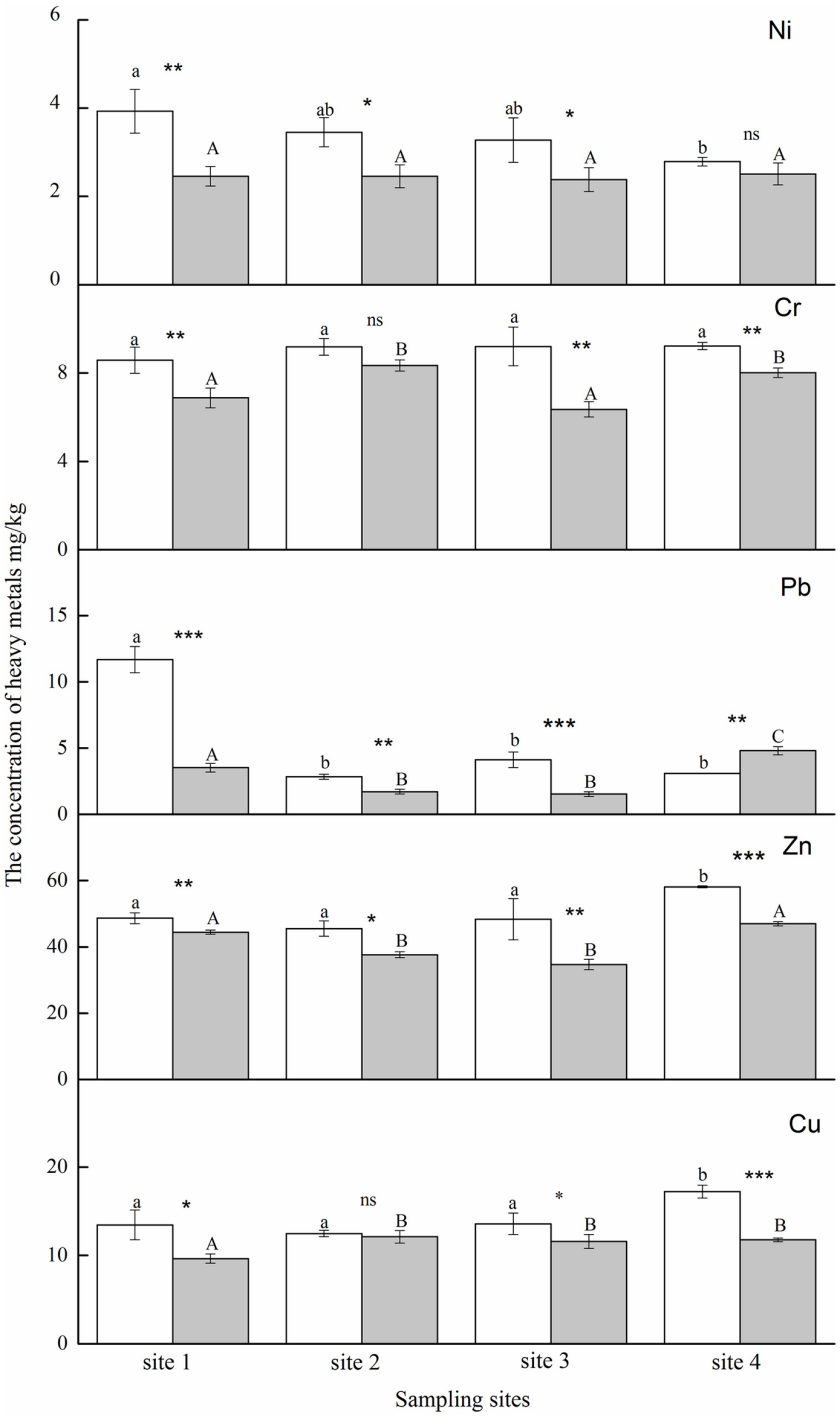
The average concentration of the investigated elements at different sampling sites during monsoon in Beijing. Gray and white bars represent park and community, respectively. Asterisks show differences between the different functional units at the same sampling sites (* *p* < 0.05, ** *p* < 0.01, *** *p* < 0.001, “ns”—not significant; *t*-test). Uppercase and lowercase letters represent park and community, respectively. Different letters indicate significant differences during the monsoon as determined by one-way analysis of variance (*P* < 0.05).

Concentrations of heavy metals on *E*. *japonicus* leaves differed between the two types of functional units (community and park; Fig 3 and Table 3). Heavy metal concentrations in communities were considerably higher than those in park areas (*P* < 0.0001 for all heavy metals; Fig 3 and Table 3). At site 2, the concentrations of Cr and Cu were not significantly affected by monsoon or functional units (*P* = 0.0578 and 0.5427, respectively). The concentrations of Ni at site 4 were not significantly different between the two types of functional areas (*P* = 0.2113). In general, the concentrations of Zn were the highest (34.700–58.088 mg/kg), whereas those of Ni were the lowest (2.456–3.929 mg/kg; Fig 3).

**Table 3:** The influence of monsoon, functional sites, and their combined effects on heavy metal concentration on *E*. *japonicus* leaves.

Element	Monsoon		Function		Monsoon & Function	
	F*	P	F	P	F	P
**Ni**	1.961	0.148	30.391	<0.0001	2.155	0.121
**Cr**	5.153	0.007	47.451	<0.0001	3.501	0.032
**Pb**	108.712	<0.0001	111.902	<0.0001	776.573	<0.0001
**Zn**	12.952	<0.0001	43.214	<0.0001	2.434	0.091
**Cu**	7.030	0.002	40.004	<0.0001	5.194	0.007

*: F: F-value (degree of freedom); P: *P*-value

During monsoon, the concentrations of heavy metals in *E*. *japonicus* leaves differed remarkably (*p* = 0.007, *p* < 0.0001, *p* < 0.0001, *p* = 0.002, respectively, for Cr, Pb, Zn, and Cu; Table 3). In community areas, the concentrations of Ni and Pb decreased during monsoon, whereas those of Zn and Cu showed the opposite tendency. Similar to that in park areas, only the concentrations of Cu showed increasing trends during monsoon, whereas the other four heavy metals showed no accurate trends. In addition, the combined effects of functional areas and monsoon were calculated (*p* = 0.121, *p* = 0.032, *p* < 0.0001, *p* = 0.091, *p* = 0.0072 for Ni, Cr, Pb, Zn, and Cu, respectively; Table 3). The effects of functional areas were greater than those of monsoon (Table 3).

Our redundancy analysis results showed that the cumulative proportion of eigenvalues from the first two axes accounted for 99.22% of the total eigenvalues, essentially reflecting all of the information. Axis1 shows the heavy metal enrichment in functional areas and accounted for 59.23% of the total eigenvalues. The values of heavy metal enrichment in community areas were positive, whereas those in the park areas were negative, indicating that functional areas were a vital factor that affected the spatial pattern of heavy metal concentrations (Table 4).

**Table 4:** Heavy metal redundancy analysis (RDA) ordination and Monte Carlo test.

Variable	Correlations with RDA ordination axes		Decision coefficient test	
	Axis1	Axis2	R^2^	*P*
**Fine**	-0.2823	0.9593	0.1029	0.219
**Large**	0.9114	0.4115	0.3820	0.002**
**Coarse**	0.7384	-0.6744	0.4347	0.003**
**TSP**	0.9534	-0.3019	0.2777	0.019*
IRHMC*	0.5923	0.6613		
CIR**	0.7131	0.9922		

* Interpretation ratio of heavy metal concentration

** Cumulative interpretation ratio between dust retention and heavy metal concentration

**Fig 4:**
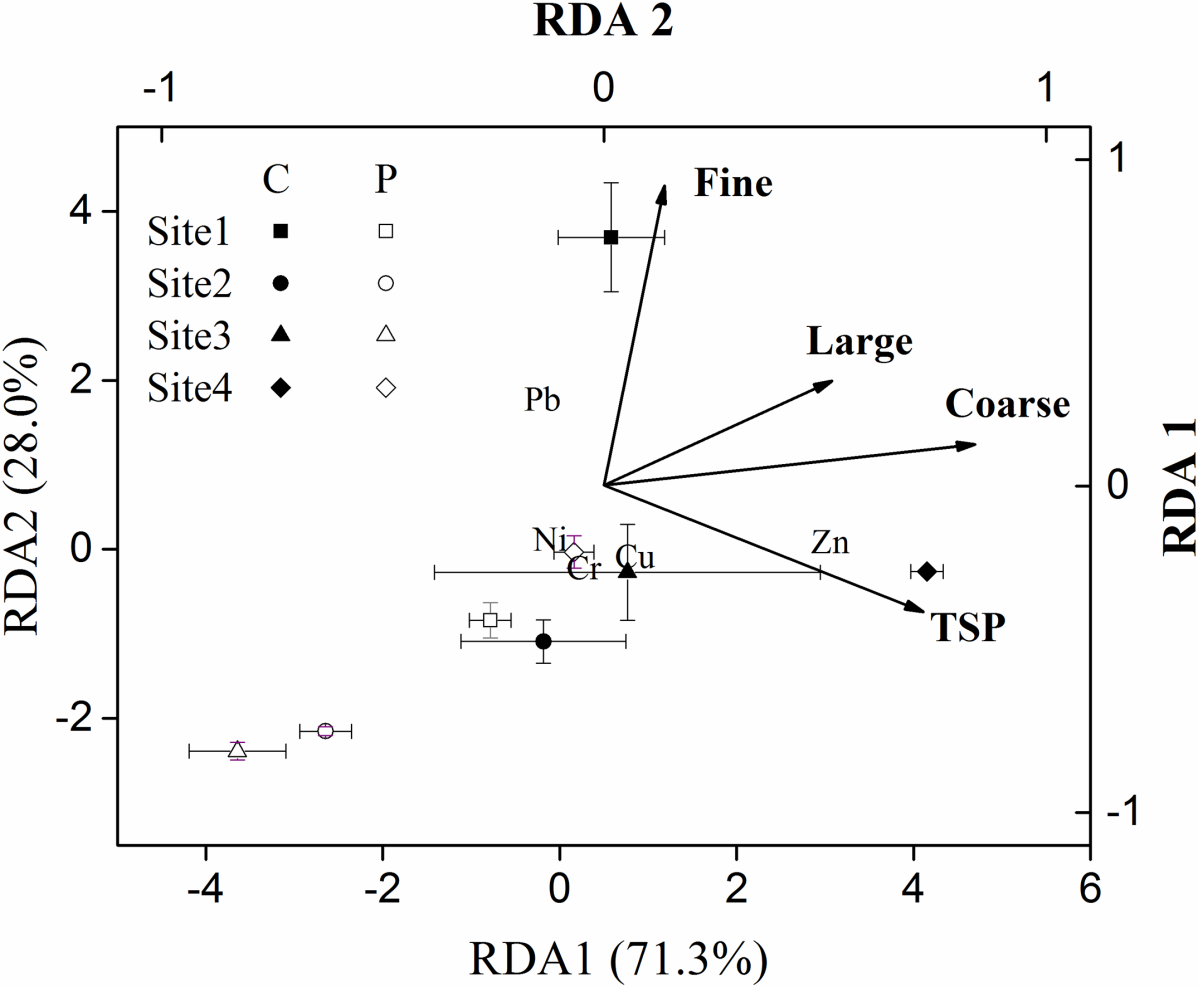
Ordination of the eight sampling sites by using redundancy analysis (RDA). Symbols refer to the sampling sites, and lines with arrows refer to the influenced factor (particulate matter). C represents community area, whereas P represents park. The abbreviation TSP and the words “Large,” “Coarse,” and “Fine” refer to total suspended particulates, large particulate matter (10 < diameter ≤ 100 μm), coarse particulate matter (2.5 < diameter ≤ 10 μm), and fine particulate matter (diameter ≤ 2.5 μm), respectively.

Our results indicated the relationship between dust retention and ordination axes and that between different particle sizes. Lines with arrows were used to indicate the dust-retention factor, and the quadrant where arrows were located indicated the positive or negative correlation between the dust-retention factor and ordination axes. The length of the arrow line indicates the magnitude of the effects on the enrichment of heavy metals of the dust-composition factor. The longer the connection, the greater would be the correlation, and clearer would be the explanation of the proportion of the distribution of heavy metals. The angles between the lines and ordination axes represent the correlation between environmental factors and ordered axis. The smaller the angle, the higher is the correlation, and vice versa (Fig 4).

In this study, the line for coarse PM was the longest and had the minimum angle with the first principal axis. Its direction with the first principal axis was the same, indicating that the first axis from left to right represented the area from parks to community areas. The accumulation of coarse PM and heavy metals showed a significant positive correlation, whereas the accumulation of total suspended, large, and coarse PM was in the same direction as the first axis. A positive correlation was noted between the concentrations of heavy metals during the transition from park to community. Further, similar to that for PM accumulation, a positive correlation existed between PM of different diameters in pairwise analysis, except for total suspended and fine PM. Determination coefficient test results showed that the retention of large, coarse, and total suspended PM was significantly related to heavy metal accumulation (*P* = 0.002, *P* = 0.003, *P* = 0.019, respectively, for large, coarse, and total suspended PM). The concentration of Zn was significantly higher in the community area, whereas the other four heavy metal concentrations were mainly higher in the park areas (Fig 4).

## Discussion

### The ability of *E*. *japonicus* leaves to capture PM

The study sites were selected by considering both the total environment influence and local ecological effect. Since no precipitation or high-speed wind occurred during the study duration, the relatively comprehensive analysis of the relationship between particulates accumulated on leaf surfaces and pollution factors could be performed.

We hypothesized that the ability of *E*. *japonicus* leaves to capture PM is influenced by the effect of functional units and monsoon, and that the influence of functional units is smaller than that of monsoon. Our results suggested that functional units had a small effect, whereas monsoon had a significant effect on the capture of PM, and the effect of monsoon showed an increasing trend from the northwest to southeast, which partly confirmed our hypothesis.

Previous studies showed that PM accumulation on leaf surfaces was strongly dependent on units [43]. Przybysz et al. [44] and Baidourela et al. [45] found that the PM retained on leaf surfaces at traffic centers was considerably higher than that in a rural area and concluded that PM retention is influenced by the district factor. However, we found that the level of PM on the leaves was less likely influenced by the functional district factor. This could be attributed to the fact that atmospheric pollution at the two functional units at the same study site was similar and, after a period of absorption, PM on the leaves reached a saturation level. Wang et al. [46] confirmed that PM retained on foliage was saturated after a certain time. *Sophora japonica* retained 3.396 g/m^2^ PM per leaf area after four weeks in a residential area of Beijing; the quantity of PM retention was similar to that obtained in our study. In contrast, in Shijiazhuang, the saturation level of PM absorbed by *E*. *japonicus* leaves was 11.620 g/m^2^, which is considerably higher than that in Beijing [3,47,48]. These differences might be attributed to the high level of pollution in Shijiazhuang, indicating that atmospheric pollution affects PM absorption.

In the present study, PM absorbed by *E*. *japonicus* leaves was found to be strongly influenced by the monsoon factor, because the inter-annual variation of haze weather in east China is associated with the East Asia monsoon, which affects the transportation of PM [49]. The PM retention on the leaves was increased from northwest to southeast, considering that the monsoon occurs in this direction.

Our study indicated that different fractions of PM absorbed by leaves were differently influenced by the monsoon. The effects of monsoon on large particulates were stronger than those on coarse particulates, whereas fine particulates were rarely influenced by the monsoon. Previous studies have shown that, during transportation, particulates with comparatively larger diameters are more likely to be influenced [50], which partly confirmed our findings.

### Heavy metals accumulated by *E*. *japonicus* leaves

Since plants differ in sizes, part of dust deposition or pollution is taken up by their roots from the soil, as well as by the leaves from air, rendering it difficult to distinguish whether the accumulated elements originate from the soil or from the atmosphere [51,52]. In this study, we could not distinguish the origin of heavy metals (from the soil or the atmosphere), and the source was uniformly regarded as from environmental pollution.

We hypothesized that the accumulation of heavy metals by *E*. *japonicus* leaves was influenced by the functional district and monsoon, and the heavy metal concentrations in the community area were considerably higher than those in the park. Site-dependent variations in PM accumulations have been reported, with higher concentration of heavy metals in polluted units than in unpolluted ones [53,54]; this finding was similar to that of our study. This could be attributed to the fact that heavy metal contamination in leaves is mainly attributed to local sources: industrial production, fossil fuel combustion, and traffic [22,26,55,56]. Analysis of the sources of heavy metals that cause pollution indicates that high percentages of Ni, Cu, Zn, As, and Cd are emitted from industrial metallurgical processes; Cr, Mn, Cu, Zn, and As from the combustion of fossil fuels; and Cu, Zn, and Cd from the abrasion of tires and brakes, whereas V, Zn, Cd, and Pb are generated from fuel additives [52,57,58]. However, determining the proportion of heavy metals taken up from the soil and that absorbed from atmospheric pollution was difficult in our study. Previous studies suggested that considerable dust deposition on leaf surfaces occurs from the air [44,59,60], and Maisto et al. [59] reported that Cr and Pb are directly taken up from the air at anthropic sites, whereas Cu is translocated from the roots to the leaves. Voutsa et al. [60] showed that trace elements in vegetable leaves originated mostly from the atmosphere. In our study, monsoon was found to have less effect on the accumulation of heavy metals than the functional district factor, indicating that the main source of heavy metals accumulated by leaves was anthropogenic, whereas the transportation from the atmosphere had less effect.

In our study, Zn and Cu contents were found to be high, whereas Pb, Cr, and Ni levels were low, which is similar to the findings reported by Liang et al. [40]. Under natural and anthropogenic conditions, the majority of plant species have been shown to accumulate considerably more Cu and Zn since they are essential microelements in all organisms and play an important role in the biosynthesis of enzymes [53,61]. Since Pb, Cr, and Ni concentrations in *E*. *japonicus* were low, it could be considered to be resistant to the hazardous effects of these heavy metals.

### Relationship between PM and heavy metal accumulation

In addition to the absorption of PM, leaves can also accumulate heavy metals. We hypothesized that a relationship exists between PM retention on leaf surfaces and heavy metals accumulated by leaves. The redundancy analysis revealed that heavy metal concentrations were positively correlated with total suspended, large, and coarse PM; this partly confirms our hypothesis. In addition, previous studies confirmed that dust retention on leaf surfaces and heavy metal accumulation by leaves was correlated [52,62,63]. Jamil et al. [64] found a significant correlation between dust retention on leaf surfaces and accumulation of heavy metals by leaves, such as Fe, Cr, Cd, Mn, and Ni, but not Cu and Zn. However, Liu et al. [65] investigated heavy metal concentrations in seven selected plants and found a negative correlation between heavy metal concentrations and PM diameter, that is, the greater the PM diameter, higher the concentrations of heavy metals. The discrepancy between their and our study findings could be attributed to the fact that PM measurement was based on PM weight, which accounts for a small proportion of PM.

Future studies need to address the following issues: (1) It would be of importance to estimate the mobility of the studied heavy metals. (2) Plant communities should be considered rather than single species. Studies should focus on large areas such as the entire North China Plain. (3) Studies should be performed over a longer time. (4) Studies should focus on the relationship between heavy metal accumulation and PM retention in or on leaves, and different fractions of particulates should be considered to obtain further accurate results.

## Conclusions

To investigate whether the PM and heavy metal retention ability of plants changed in different human-disturbance areas along monsoon directions in Beijing, we used rinse-and-weigh method and electro-thermal atomic absorption spectrometry to measure the amount of PM and heavy metals such as Ni, Cr, Cu, Pb, and Zn accumulated by the leaves of *E*. *japonicus*. Our results revealed that the types of functional units were found to exert little influence on the capture of PM, whereas monsoon had a significant effect, showing an increasing trend from the northwest to southeast (0.005, 0.453, 0.643, and 1.569 g/m^2^ for fine, coarse, large, and total PM, respectively, to 0.015, 2.687, 1.941, and 1.569 g/m^2^, for fine, coarse, large, and total PM, respectively). Functional district-dependent variations were found in heavy metal accumulation, and heavy metal accumulation was higher in communities than in parks. Moreover, a positive relationship was found between PM retention and heavy metal accumulation by the leaves of *E*. *japonicus*. Taken together, our results suggested that the PM and heavy metal retention ability of *E*. *japonicus* was sensitive to human disturbance and monsoon in Beijing. Since *E*. *japonicus* is a widely distributed tree and has the ability to purify the atmosphere, it might play an important role in mitigating urban environmental pollution.
